# Influence of Type of Cross-Linking Agent on Structure and Transport Properties of Polydecylmethylsiloxane

**DOI:** 10.3390/polym15224436

**Published:** 2023-11-16

**Authors:** Evgenia Grushevenko, Tatiana Rokhmanka, Stepan Sokolov, Andrey Basko, Ilya Borisov, Konstantin Pochivalov, Alexey Volkov

**Affiliations:** 1 A.V. Topchiev Institute of Petrochemical Synthesis, Russian Academy of Sciences, Leninsky Prospect 29, 119991 Moscow, Russia; rokhmankatn@ips.ac.ru (T.R.); sokolovste@ips.ac.ru (S.S.); boril@ips.ac.ru (I.B.); 2 G.A. Krestov Institute of Solution Chemistry, Russian Academy of Sciences, Akademicheskaya 1, 153045 Ivanovo, Russia; avb@isc-ras.ru (A.B.); pkv@isc-ras.ru (K.P.)

**Keywords:** polydecylmethylsiloxane, cross-linking, correlation structure and properties, hydrocarbon separation

## Abstract

The development of membrane materials with high transport and separation properties for the removal of higher hydrocarbons from gas mixtures is an important and complex task. This work examines the effect of a cross-linking agent on the structure and transport properties of polydecylmethylsiloxane (C10), a material characterized by high selectivity towards C_3+_ hydrocarbons. C10 was cross-linked with various diene hydrocarbons, such as 1,7-octadiene (C10-OD), 1,9-decadiene (C10-DD), 1,11-dodecadiene (C10-DdD), and vinyl-terminated polysiloxanes, of different molecular weights: 500 g/mol (C10-Sil500) and 25,000 g/mol (C10-Sil25-OD). Using a number of characterization methods (IR-spectroscopy, WAXS, DSC, toluene sorption, and gas permeability), it was revealed that a change in the type and length of the cross-linking agent (at the same mole concentration of cross-linking agent) led to a significant change in the structure of the polymer material. The nature of cross-linking agent affected the arrangement of the decyl side-groups of the polymer, resulting in noticeable differences in the solubility, diffusivity, permeability, and selectivity of tested gases (N_2_, CH_4_, C_2_H_6_, and C_4_H_10_). For instance, an increase in the length of the hydrocarbon cross-linker was associated with a drop of n-butane permeability from 5510 (C10-OD) to 3000 Barrer (C10-DdD); however, the transition to a polysiloxane cross-linker led to an increase in corresponded permeability up to 8200 Barrer (C10-Sil25-OD). The n-butane/nitrogen selectivity was significantly higher for diene-type cross-linkers, and the maximum value was achieved for 1,7-octadiene (α(C_4_H_10_/N_2_) = 104).

## 1. Introduction

In membrane materials science, much attention has been paid to determining the structure–transport properties correlation of polymer materials. With regard to the creation of polymer membranes, it is important to develop a selective polymer that is both highly permeable and highly selective, which is a rather difficult task. Much attention is paid to creating a specific polymer structure that determines its outstanding transport properties [1]. Examples of such approaches are the introduction of selective side groups into the polymer chain [2,3,4], copolymerization [5,6], the introduction of specific particles [7,8,9], the directed task of organizing the membrane material [10,11,12], etc.

Siloxane rubbers (primarily polydimethylsiloxane (PDMS)) are widely known and actively used membrane materials due to their high permeability, even at low temperatures and pressures. An important feature of membranes based on siloxane rubbers is their cross-linked structure, which ensures the mechanical stability of the membranes in a volatile organic compound (VOC) environment (non-cross-linked polymers may partially dissolve in VOC-containing media, which significantly reduces their performance properties). Rubber-like PDMS has prevailed since the late 20th century, as it is a highly permeable and selective material used for VOC/N_2_ separation [13,14,15,16,17,18]. Most organoselective commercial membranes are based on silicone rubbers. These materials have earned widespread popularity due to their ease of use, separation properties, and chemical and thermal resistance. The development of highly selective membranes based on them is an urgent task. PDMS exhibits high permeability and selectivity for VOC vapors. However, it is known that when moving from individual substances to mixtures, the VOC selectivity of silicone rubbers noticeably decreases [19]. This effect is associated with the significant swelling of PDMS in VOCs and an increase in the diffusion of a small component (gas) through the membrane. The transition from PDMS to polysiloxanes substituted on the main or side chain allows increasing selectivity towards organic vapors [3]. However, the decrease in the selectivity characteristic of PDMS when moving from individual components to a mixture remains for side-chain-substituted polysiloxanes [20,21]. It should be noted that one of the approaches for reducing the swelling of polysiloxanes in the mixture being separated is chemical cross-linking. Before cross-linking, polysiloxan is a viscous liquid. Chemical cross-linking makes it possible to obtain a continuous polymer film. Thus, the effect of the polymer–cross-linking agent ratio of the two-component system KE-1310 (PDMS, Shin-Etsu Polymer Co., Ltd., Tokyo, Japan) on the separation of toluene, m-xylene, and methanol from a mixture containing nitrogen was studied in [18]. When varying the polymer:cross-linker ratio from 10:1 to 10:5, the extreme dependences of the toluene flux and separation factor were observed, with a maximum at a ratio of 10:4. Depending on the cross-linking, the efficiency of toluene recovery varied from 63 to 74%. However, the authors of the article considered this difference to be insignificant, and the effect of cross-linking on the separation properties of membranes was not considered in more detail. Most studies in the field of VOC vapor recovery using membranes have considered the influence of process conditions and the nature of the penetrant, but very few studies have been devoted to the influence of the degree of cross-linking on the transport and separation properties of the membrane. This area has great potential in terms of varying membrane properties, in particular for controlling its swelling in VOCs, which leads to an undesirable decrease in selectivity. Studying work devoted to the production of PDMS membranes under various conditions of the cross-linking process can be considered a prerequisite in this area. For example, the decrease in the methane permeability coefficient of PDMS from 1000 to 480 Barrer is associated with an increase in the cross-linking reaction temperature from 75 to 100 °C, with a decrease in the fraction of free volume in the polymer (at 0.4 bar) being recorded [22]. To obtain continuous membranes with the required mechanical characteristics, cross-linking agents with various functional groups are used. The most common cross-linking of PDMS with alkoxysilanes in the terminal silanol groups of the polymer occurs in the presence of tin catalysts [12,23,24]. Thus, increasing the tetraethoxysilane content in the PDMS reaction mixture from 0 to 30 wt% leads to a decrease in the oxygen permeability coefficient from 640 to 185 Barrer (at 0.5 bar) in work [12]. We noted the relationship between the cross-link density of octyl-grafted poly(hydromethylsiloxane), the type and amount of cross-linking agent used (poly(hydromethylsiloxane) or poly(octylmethylsiloxane)), and the transport properties [25]. We also noted that the introduction of a larger amount of poly(octylmethylsiloxane) leads to a decrease in the cross-link density and an increase in the permeability and selectivity of the material for VOC vapors. It was demonstrated that the use of a shorter cross-link made it possible to increase the selectivity of n-butane with respect to methane while maintaining the permeability of n-butane at a comparable level [26].

So, the cross-linked polyorganosiloxanes chemical structure of cross-linking agents also affects the structure and transport properties of the membrane material. In this regard, in this work, the effects of the type of cross-linking agent used for polydecylmethylsiloxane on its structure and gas transport properties were studied for the first time. The fundamental novelty of this work lies in the determination of the relationship between the type and length of cross-linking and the transport properties of comb-shaped polysiloxanes, which are mainly determined based on the resulting supramolecular structure of the polymer.

## 2. Materials and Methods

### 2.1. Materials

The reagents used for polymer synthesis were polymethylhydrosiloxane (PMHS) (M_n_ = 1900 g/mol, ABCR, Karlsruhe, Germany); 1-decene (95 wt%, Sigma-Aldrich, St. Louis, MO, USA); isooctane (chemical grade, Component Reactiv, Moscow, Russia); 1,7-octadiene (95 wt%, Sigma-Aldrich); 1,9-decadiene (98 wt%, Alinda Chemical, Moscow, Russia); 1,11-dodecadiene (99 wt%, Alinda Chemical); 1,3-divinyl-1,1,3,3-tetramethyl disiloxane platinum complex (0), a solution in xylene (Sigma-Aldrich); polydimethylsiloxane vinyl terminated (PDMS) with a different molecular weight of M_n_ = 25,000 g/mol (Sigma-Aldrich); and M_n_ = 500 g/mol (ABCR, Karlsruhe, Germany). Chemical-grade toluene (Component Reactive, Moscow, Russia) was used for sorption measurement. For the characterization of membrane transport properties, the following individual gases were used: C_4_H_10_ (99.5% vol., Monitoring, Saint-Petersburg, Russia), C_2_H_6_ (99.5% vol., Monitoring), CO_2_ (99.5% vol., MGPZ, Moscow, Russia), N_2_ (99.5% vol., MGPZ), and CH_4_ (99.5% vol., MGPZ).

### 2.2. Synthesis Procedure

**Figure 1:**
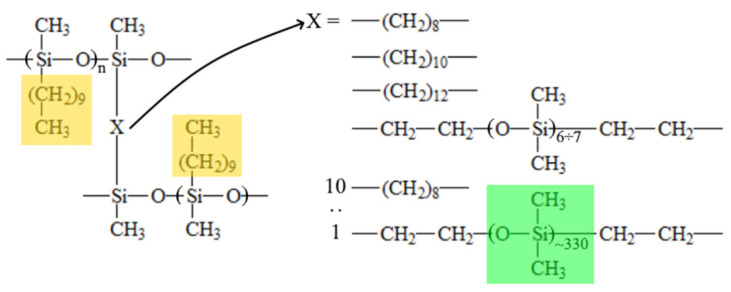
The schematic structure of cross-linked polydecylmethylsiloxanes.

The polymer was obtained according to the procedure described in [27] via the hydrosilylation reaction. The PMHS was mixed with a 15 wt% solution of 1-decene in isooctane in the presence of Carstead catalyst (1,3-divinyl-1,1,3,3-tetramethyl disiloxane platinum (0) complex), and the solution in xylene was stirred for 2 h at 60 °C. Then, the solution of the cross-linking agent (mole ratio of 1-decene/cross-linking agent = 15–20) in isooctane was added to the reaction mixture. The types and concentrations of the cross-linking agent (CA) are represented in Table 1. The stirring of the reaction mixture continued for one hour. After that, 3 wt% PMHS solution in isooctane was added to the reaction mixture to the stoichiometric ratio. The schematic structure of cross-linked polydecylmethylsiloxanes is shown in Figure 1. Membranes were obtained by casting a polymer solution on a Teflon surface, with subsequent drying at 60 °C until it reached a constant weight. The membrane thickness was 100 ± 10 µm.

### 2.3. Study of the Physicochemical Properties of the Polymer

The attenuated total reflection (ATR) infrared (IR) spectra of the membrane samples were recorded using a ZnSe crystal (scan. −50, resolution 2 cm^−1^) via an IFS-66 v/s Bruker spectrometer (Billerica, MA, USA). The detection range was 600–4000 cm^−1^.

Calorimetric measurements used to obtain thermograms of polymer samples were performed using a DSC 3+ differential scanning calorimeter (Mettler Toledo, Greifensee, Switzerland) in an argon atmosphere. The temperature range extended from −140 to 100 °C, and the scanning speed was 10 °C/min with an accuracy of 0.05 °C.

The X-ray diffraction spectra were recorded via the use of a Rigaku Rotaflex RU-200 apparatus equipped with a rotating copper anode (Cu Kα radiation, Ni filter). The apparatus operating modes were 30 kV and 100 mA. A Rigaku D/MAX-RC horizontal wide-angle goniometer was used for the X-ray photography of the samples. Imaging was performed according to the Bragg–Brentano scheme in the *θ*–2*θ* geometry. The angle scanning range was from 5 to 55° at 2*θ*. The scanning speed was 2°/min in 0.04° increments. The detector for the diffracted X-ray emission was a scintillation counter. The measurement temperatures were 20 °C and −190 °C. A diffraction pattern of a sample cooled to the temperature of liquid nitrogen (−196 °C) was obtained using a special low-temperature attachment. Membrane samples, each with a thickness of 500 μm, were mounted on a vertical copper table. The diffraction patterns were processed utilizing the Fityk program: background noise was subtracted, and the patterns were approximated by employing the deconvolution technique with the sum of multiple Gaussian peaks [28]. Peak approximation was carried out with an accuracy of 97%. Interplanar distances were determined using the Wulff–Bragg formula, which calculates the value through the 2*θ* peak angular position. In this formula, Å represents the wavelength, and *θ* denotes the glancing angle:

(1) λ=2dsinθ

The fraction of crystalline material formed by side substituents (colored with yellow in Figure 1) or the crystallinity degree (*α*) can be calculated [29] using Formula (2):

(2) $$\alpha =A_{c}/A_{sum}\;C=A_{c}/A_{sum}$$

where *A_c_* represents the total sum of peak integral intensities (areas) that correspond to phase *C*, and *A_sum_* denotes the total area of all peaks used for approximating the diffraction pattern. The accuracy of determining the degree of the crystallinity of the polymer was 15%.

The density of polymer films was calculated based on the data obtained via the hydrostatic weighing method, as described in detail in [30]. Using an electronic microbalance (Sartorius analytic A120S, Göttingen, Germany), the mass of the sample was determined in air (*m_0_*) and water as a non-wetting liquid (*m*). Then, the density of samples was determined using Formula (3):

(3) $$\rho =\frac{m_{0}\cdot \rho_{H_{2}O}}{m_{0}-m}$$

The membrane samples’ cross-link densities (*γ_e_*) were computed through the utilization of the Flory–Rehner equation [31] based on the data regarding sample swelling in toluene [32]:

(4) $$\gamma_{e}=-\frac{\ln\left(1-\nu_{2}\right)+\nu_{2}+\mu \nu_{2}^{2}}{V_{1}\times \left(\nu_{2}^{\frac{1}{3}}\times \nu_{0}^{\frac{2}{3}}-\frac{\nu_{2}}{2}\right)}$$

where *V_1_* is the molar volume of the solvent (107·10^−3^ L/mol, toluene), ν_2_ is the volume fraction of the polymer in the swollen cross-linked rubber (can be equal to the reciprocal of the equilibrium swelling volume), *ν_0_* is the dissolution taken into account as the volume fraction of the polymer in the formed rubber, and *µ* is the polymer–solvent interaction parameter.

To determine polymer sample sorption and swelling in toluene, we employed continuous membranes, each with a minimum thickness of 100 μm. To do so, the mass and geometric dimensions of the membrane samples were measured before and after being kept in toluene for 72 h [33]. Before measurements, excess toluene was carefully removed from the surface using filter paper. The sorption (*S*) and swelling degree (*Q*) were calculated using Equations (5) and (6) as follows:

(5) $$S=\frac{m_{s}-m_{i}}{m_{i}}$$

(6) $$Q=\frac{V_{s}-V_{i}}{V_{i}}$$

where *m* is the mass of polymer sample, *V* is the volume of polymer sample, and indices *i* and *s* are the original sample and the sample after 48 h of exposure in toluene.

### 2.4. Study of the Gas Transport Properties of Polymer Film

The gas permeability and diffusion coefficients of membrane materials for individual gases (N_2_, CO_2_, CH_4_, C_2_H_6_, C_4_H_10_) were measured at 30 °C according to the Daynes–Barrer standard technique using the precise unit “Helmholtz-Zentrum Geesthacht” mounted with a pressure sensor (“Baratron”), with an accuracy of 10^−7^ bar. Permeability was measured in Barrer. The pure gas permeability coefficient *P_i_* was determined via the variable pressure/constant volume method, the diffusivity *D_i_* was measured according to the time-lag method, and the solubility coefficient *S_i_* was estimated as $S_{i}=P_{i}/D_{i}$. The ideal selectivity for the gas pair n-butane and methane is given by the following relationship:

(7) $$\alpha =P_{C_{4}H_{10}}/P_{{\mathrm{CH}}_{4}}=\left(D_{C_{4}H_{10}}/D_{{\mathrm{CH}}_{4}}\right)\cdot \left(S_{C_{4}H_{10}}/S_{{\mathrm{CH}}_{4}}\right)=\alpha_{D}\cdot \alpha_{S}$$

where $\alpha_{D}$ is the diffusivity selectivity, and $\alpha_{S}$ is the solubility selectivity. The experimental error of the measurements of *P* was 5%, and that of *D* was 9%.

## 3. Results and Discussion

### 3.1. Structure and Physico-Chemical Properties of Cross-Linked Polydecylmethylsiloxanes (C10)

#### 3.1.1. IR-Spectroscopy

**Figure 2:**
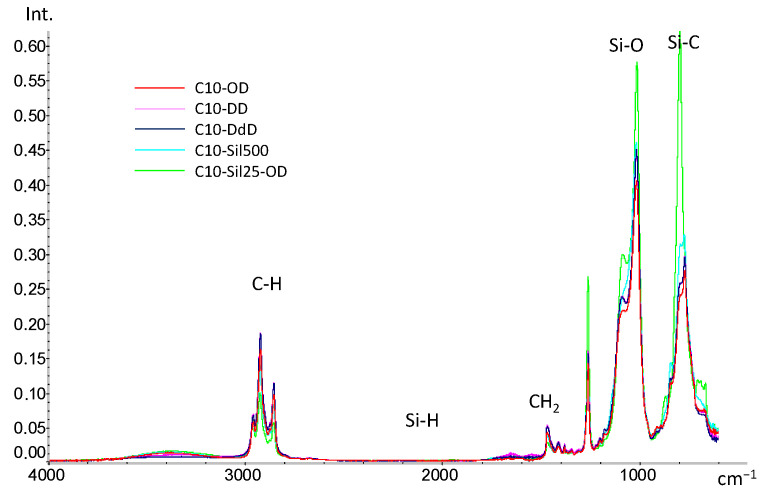
IR-spectra of cross-linked C10.

Survey IR spectra (Figure 2) obtained for cross-linked C10 samples confirm the occurrence of the hydrosilylation reaction: the bands at 2168 cm^−1^, corresponding to the Si-H bond, are completely absent in all spectra. The absence of the characteristic spectral features of double bonds from olefins (bands above 3000 cm^−1^ from =CH_2_, 1640–60 cm^−1^ from C=C and 910 cm^−1^ from bending vibrations at the CH=CH_2_ node) indicates the addition of introduced cross-linking agents to both double bonds.

**Figure 3:**
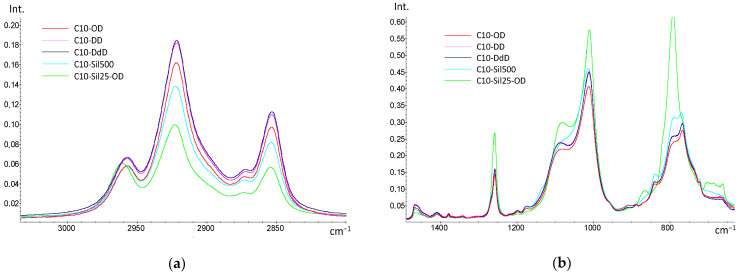
Detailed IR spectra of cross-linked samples C10: (**a**)—2800–3000 cm^−1^; (**b**)—750–1450 cm^−1^.

In the spectra of all samples, saturated -CH_2_- groups are clearly visible (bands 2840–2960 cm^−1^—ν_CH_, and bands 1380–1460 cm^−1^—δ_CH_). It is worth noting that when detailing the region of 2840–2960 cm^−1^, it becomes noticeable that with an increase in the -CH_2_- groups in the introduced cross-linking agent (in the case of diene hydrocarbons) and a decrease in the length of the siloxane chain (in the case of vinyl-terminated polysiloxanes), the intensity of the bands increases (Figure 3a). Differences between cross-linked samples are also observed in the region of 800–1100 cm^−1^ (Figure 2b). Thus, compared to the spectrum of the original PMGS [27], the bands from the Si-O (1000–1100 cm^−1^) and Si-C (900–800 cm^−1^) bonds strongly shift to the long wavelength region, which is a direct indication of the emergence of new Si-C bonds and the appearance of cross-links in the siloxane chain. The differences in the observed intensity of the bands can be associated with the different natures of cross-links in the C10 macromolecule: the lowest intensity is characteristic of samples cross-linked with diene hydrocarbons, and the highest is characteristic of the C10-Sil25-OD sample cross-linked with PDMS. Since the cross-linking of PDMS leads to a quantitative increase in the Si-O and Si-C bonds in the polymer, an increase in the intensity of the corresponding bands is also observed.

#### 3.1.2. Differential Scanning Calorimetry (DSC)

**Figure 4:**
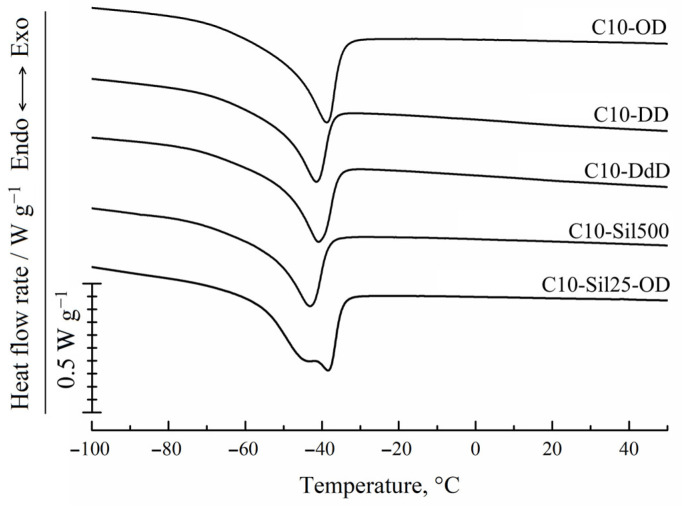
DSC-curves for cross-linked C10 obtained in heating mode at a rate of 10 °C/min sample.

The results of the DSC examination of the samples are illustrated in Figure 4. It can be seen that in the DSC thermograms of samples C10-OD, C10-DD, C10-DdD, and C10-Sil500 obtained in the heating mode, there are unimodal peaks, and in the thermogram of sample C10-Sil25000, there is a bimodal endothermic peak. The temperatures of the maximum values of these peaks are given in Table 2. Taking into account the data used in [27,34,35], we can conclude that the endothermic peak in this temperature range (Table 2) reflects the thermal effect of melting crystallites formed by side alkyl substituents (highlighted in yellow in Figure 1). In works devoted to uncross-linked polyalkylsiloxanes [34] and polythioalkylsiloxanes [35], it was shown that side substituents crystallize to form a hexagonal crystal lattice with a characteristic interplanar distance of 4.0–4.1 Å, characteristic of n-alkanes. This fact is in good agreement with the assumption made above that the crystallization process of all samples except C10-Sil25000 exclusively involves the side substituents of the polymer chain (fragments highlighted in yellow in Figure 1). It should also be noted that the data obtained in this work for cross-linked C10 correlate well with the previously published results [27]. It can be seen that glass transition does not appear in the obtained DSC curves, and the melting temperature of the samples differs slightly and varies within ±2 °C (Table 2).

Considering that in the C10-Sil25-OD sample, the cross-linking agent (vinyl-terminated PDMS) itself is a polymer capable of crystallization, the bimodality of the endothermic peak in the thermogram of this sample gives reason to believe that it contains not only crystallites formed by side C10 substituents (shown in yellow in Figure 1) but also crystallites formed by a polymer cross-linking agent (shown in green in Figure 1), consisting of an average of 330 units. Obviously, the formation of crystallites from the units of a single cross-link is unlikely. Therefore, the condition required for the formation of PDMS crystallite nuclei is the concentration of high-molecular cross-linking units in some microregions of the sample. The formation of another type of crystallites in the sample under discussion is also shown by the higher melting enthalpy compared to other samples (Table 2).

In Figure 4 and Table 2, it is clear that the temperature at the maximum high-temperature peak in the thermogram of the C10-Sil25-OD sample practically coincides with the temperature of the peak maximum in the thermogram of the C10-OD sample. Considering that only 10% of the total number of cross-links in the C10-Sil25-OD sample have a structure different to the structure of the cross-links in the C10-OD sample, it can be assumed that it is the high-temperature part of the bimodal peak that reflects the thermal effect of melting crystallites formed by side C10 substituents (shown in yellow in Figure 1), and low temperature is the thermal effect of melting PDMS crystallites. This assumption agrees well with data regarding the melting temperature of the PDMS homopolymer [36].

As shown in Figure 4 and Table 2, the peak area (melting enthalpy) in the thermograms of the other samples is close, and only for the C10-OD sample is it noticeably larger. This result is unexpected, since, in the case of the sample cross-linked with 1,7-octadiene, the cross-link length is less than the length of the side C10 substituent (see Figure 1), and, therefore, the crystallization process of the side fragments should have experienced steric hindrances. An explanation of this issue will be given below using additional experimental data.

#### 3.1.3. Wide Angle X-ray Scattering (WAXS)

**Figure 5:**
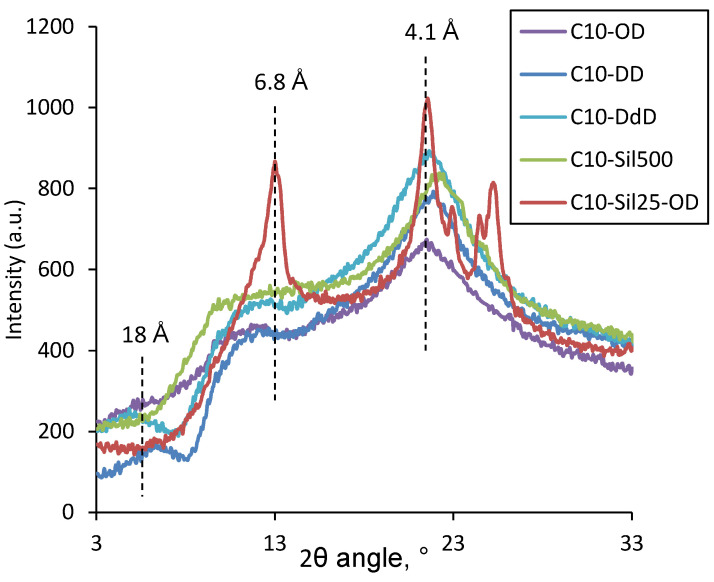
WAXS curves for cross-linked C10 at low temperature (−196 °C).

To confirm the validity of the conclusions drawn from the analysis of the DSC curves, WAXS data were obtained at temperatures below the melting point (Figure 5). At room temperature, polysiloxanes are amorphous, and the resulting diffraction pattern will not be informative. Based on the data obtained in an atmosphere of liquid nitrogen (−196 °C), one can judge the trends in the degree of crystallinity with regard to changes in the cross-linking agent.

Figure 5 shows that for all the resulting cross-linked C10 samples, a peak is observed in the 2*θ* region at around 20°. This peak corresponds to an interplanar distance of ~4.1 Å, which, taking into account the data of the classical work of Plate and Shibaev [37], confirms the above conclusion regarding the presence in the samples of crystallites formed by the ordering of side-chain substituents. The C10-Sil25-OD sample exhibits a diffraction pattern characteristic of the crystallites of similar PDMS [27]. The reflection peaks near 2*θ* > 20° correspond to PDMS backbone crystallization (3.5, 3.6, and 3.9 Å) [38]. Nevertheless, for C10-Sil25-OD, due to combined cross-linking, the following signs of polydecylmethylsiloxane cross-linked with 1,7-octadiene appear: a peak characteristic of the interplanar distance in the crystallites of side-chain substituents; a 10–15° plateau, related to the interplanar distance of bridges of cross-links (9–11 Å); and polymer chains, separated by side chains (8–18 Å). Such a plateau is observed for all cross-linked C10 considered in this work and can be decomposed into a series of Gaussian peaks. The above is fully consistent with the assumption made above regarding the reasons for the appearance of the bimodality of the peak in the DSC thermogram of the C10-Sil25-OD sample. The values of interplanar distances obtained via calculation are presented in Table 3.

The interplanar distances d_1_, d_2_, and d_3_ describe the cross-linking and ordering of the siloxane chains of polydecylmethylsiloxane. Based on the analysis of the data in Table 3, it can be concluded that the use of the cross-linking agent 1,9-decadiene leads to the formation of an ordered structure in polydecylmethylsiloxane with the smallest interplanar distance d_1_, which can be associated with polymer chains, separated by side decyl substituents. Moreover, one of the shortest interplanar distance of bridges of cross-links was found for this type of cross-linking agent. The calculation of the lengths of hydrocarbon radicals (octyl—11.9 Å, decyl—14.4 Å, dodecyl—17.0 Å) suggests that the distance d_1_ corresponds to the distance between neighboring decylsiloxane chains. The difference in the d_1_ value in this case can be attributed to differences in the length of the hydrocarbon cross-link and the side group. Interplanar distances d_2_ and d_3_ can be attributed to different stitching orientation options. In the case of siloxane cross-links, the characteristic interplanar distance d_1_ is absent. This may be due to both the large size of the cross-link itself (as in the case of C10-Sil25-OD) and the greater mobility of the siloxane chain, as a result of which the ordering of neighboring chains in the plane is not observed. Taking into account the mathematical processing of WAXS data, the mass fraction of crystallites formed by lateral substituents was calculated, determining the crystallinity degree of side-chain substituents (*α*) in the samples (Table 3). It can be seen that with an increase in the length of the hydrocarbon cross-link, *α* increases from 5.7 (OD) to 6.9% (DdD), which is apparently due to a decrease in the steric hindrance for crystallization. This assumption is perfectly complemented by data for siloxane cross-links, for which the degree of decyl substituents’ crystallinity was 7.1% (C10-Sil500) and 7.6% (C10-Sil25-OD), respectively. It should be noted that the trend obtained from WAXS data does not agree with the data obtained via DSC, according to which the C10-OD sample has the highest melting enthalpy (ΔH), and, consequently, the decyl substituent crystallinity degree. This is probably due to the fact that the samples were cooled before DSC and WAXS experiments under different conditions: in the DSC experiment, cooling was carried out at a rate of 10 °C/min, which left enough time for the necessary rearrangements in the structure of the sample, while during preparation, samples used for X-ray analysis were cooled via almost instantaneous freezing in liquid nitrogen.

#### 3.1.4. Cross-Linking Density

An important characteristic of cross-linked polymers is the cross-link density (*γ_e_*). This value is determined from data on the swelling of cross-linked polymers in toluene. Table 4 shows the values of toluene sorption (*S*), degree of swelling (*Q*); density (ρ); cross-link densities (*γ_e_*) calculated using Equation (4).

Taking into account the IR spectroscopy data, according to which there are no unreacted components in the samples, the average molecular weight between adjacent cross-links can be easily calculated based on the 1-decene/CA ratio indicated in Table 1 (15 and 20). For samples cross-linked using dienes and PDMS, this value is ~2000 and 1500 g/mol, respectively, which corresponds to a cross-link density of 5.00 and 6.67·10^−4^ mol/g. This means that the apparent cross-link density values calculated from swelling data characterize not only the average number of chain links between cross-links but also the ability of cross-links of different natures to limit the swelling of the sample.

Based on the data given in Table 4, it is clear that the density of the samples decreases with the increasing length of the cross-linking agent in the series of dienes (OD, DD, and DdD). Although, according to WAXS data, an increase in the cross-link length leads to an increase in the degree of crystallinity (at a low temperature), it can be assumed that in the amorphous state, a decrease in the cross-link length leads to the convergence of C10 molecules (see the d_1_ value in Table 3) and, consequently, the compaction of side substituents. The density of samples cross-linked using even longer siloxane cross-linkers was found to be comparable to that of the C10-DdD sample.

It is also shown in Table 4 that the apparent cross-linking density of all samples calculated using the Flory–Rehner equation is significantly lower than that calculated based on the ratio of the amounts of 1-decene and the cross-linking agent during synthesis. In our opinion, this situation is as expected, given that even when low-molecular cross-linking agents are used, the lengths of cross-links are comparable to the lengths of several segments of the main chain. In addition, it can be seen that with the increasing length of the cross-linking agent, the degree of swelling of the samples increases, and the apparent cross-link density decreases. This is obviously due to the fact that with increasing cross-linking length, the structure becomes looser (which is consistent with the data regarding the density of the samples), and the free volume between neighboring C10 macromolecules available for toluene sorption increases. When moving from diene cross-linkers to even longer vinyl-terminated PDMS, the degree of swelling continues to increase, and the apparent cross-link density decreases. Moreover, the change in these characteristics is disproportionate to the increase in the cross-link length (for example, for C10-Sil500, the cross-link length is ~25.2 Å, which is less than one and a half times greater than that in the case of C10-DdD, while the degree of swelling almost doubles). Considering the differences in the size of the Kuhn segment for polyethylene (20.8) and PDMS (14), this fact can be associated with an increase in the cross-linking flexibility. More flexible siloxane cross-linking places fewer restrictions on the sample swelling process. This assumption is also confirmed by a slight increase in the degree of side-chain substituents’ crystallinity, according to low temperature WAXS data and the absence of a peak corresponding to the d_1_ size in the diffraction pattern, which characterizes the spatial orientation of neighboring C10 macromolecules, which is easily disrupted when the flexibility of the cross-linking agent is sufficiently high. Increasing the length of the siloxane cross-linking agent (C10-Sil25) naturally leads to a further decrease in the apparent cross-link density and an increase in the degree of swelling, despite the fact that the actual cross-link density of these samples is approximately 33% lower (Table 1). These changes, however, are not so large, which is probably due to the fact that a significant proportion of the mass of the C10-Sil25 sample is directly occupied by PDMS cross-links, which are less prone to swelling in toluene due to the lower thermodynamic affinity of PDMS-toluene. The picture changes with the introduction of combined (hydrocarbon and siloxane) cross-linking. Thus, for the C10-Sil25-OD sample, a 20% higher apparent cross-link density was obtained than for its analogue without 1,7-octadiene (C10-Sil25). The density and degree of swelling of the sample in toluene decrease with the addition of a second cross-linking agent—1,7-octadiene. Apparently, the combination of a short rigid hydrocarbon cross-link and a long mobile siloxane cross-link leads to the formation of two types of clusters in the polymer network: polydecylmethylsiloxane, the side groups of which are fixed through rigid cross-linking, and a polydecylmethylsiloxane-polydimethylsiloxane copolymer.

### 3.2. Gas Transport Properties of Cross-Linked Polydecylmethylsiloxanes

The structures of polymers also determine their transport properties. In this regard, for the synthesized cross-linked polydecylmethylsiloxanes, the diffusion coefficients (Table 5), namely solubility (Table 6) and permeability (Table 7) for individual gases (nitrogen, carbon dioxide, methane, ethane, n-butane), are considered.

The diffusion coefficients for C10 cross-linked with diene hydrocarbons and siloxanes differ. The presence of a flexible siloxane cross-link leads to high diffusion coefficients for all the gases studied. As the length of the siloxane cross-link increases, the diffusion coefficients of nitrogen increase. It is worth noting that if, in the case of nitrogen transfer through a polymer film, the exclusive influence of the cross-link length is noticeable, when moving to larger gases, a correlation is observed between the apparent cross-link density and the diffusion coefficients. Thus, for methane, the diffusion coefficient is higher for the C10-Sil500 sample than for the C10-Sil25-OD sample. Thus, the effect of the addition of 1,7-octadiene leads to a decrease in the diffusion permeability of C10 cross-linked long-chain PDMS for hydrocarbons. In the series of diene cross-linking agents, the diffusion coefficients of nitrogen and methane pass through a local minimum (C10-DD). This effect can be associated with two factors: the domains formed by side groups have reduced diffusion permeability (a characteristic trend is observed for ethane and n-butane); in contrast, the areas near the cross-links, depending on the type and length of the cross-linking agent, are accessible for diffusion and have different sizes. Thus, it can be assumed that when the lengths of the cross-linking agent and the side group are similar (as in the case of C10-DD), the size of the region near the cross-links is minimal, which is indirectly confirmed by decreases in the diffusion coefficients of nitrogen and methane. The DSC data are in good agreement with this assumption: the highest enthalpy of melting was found for sample C10-OD, for which the cross-link length was two methylene groups less than the length of side group. This, in turn, leads to smaller interplanar distances and concentrations of side group crystallites, according to WAXS data. The highest value of the apparent cross-linking density also puts limitations on the mobility of the macromolecules of the C10 chain. The presence of such a strained cross-link for the polymer under study apparently leads to the formation of “voids” in the cross-link area, which, in turn, ensure that the C10-OD sample achieves values comparable to C10 cross-linked with flexible siloxane cross-links. For ethane and n-butane, with the increasing length of diene cross-linking agents, diffusion coefficients increase, which correlates well with a decrease in the apparent cross-linking density of the polymers.

The structural features of the resulting cross-linked C10 are clearly demonstrated when considering the selectivity of diffusion of hydrocarbon penetrants with respect to nitrogen. There is a direct correlation between diffusion selectivity and the apparent cross-linking density: the higher the apparent cross-linking density, the greater the diffusion coefficient ratio.

A general tendency towards increases in the solubility coefficients of gases in the polymer with increasing length of the cross-linking agent was identified, which correlates well with the decrease in the density of the polymer in this series (Table 4). As can be seen from Table 6, the solubility coefficients of n-butane and ethane for synthesized polydecylmethylsiloxanes are an order of magnitude higher than the similar values for methane and nitrogen. At the same time, the high selectivity of solubility of C_2+_ hydrocarbons relative to nitrogen in these materials makes a decisive contribution to the overall selectivity of the separation of these gases. It is interesting to note that among linear hydrocarbon cross-linking agents, the highest solubility values of ethane and n-butane in polydecylmethylsiloxanes are observed in the case of 1,9-decadiene (sample C10-DD). Apparently, this can be interpreted based on the similar lengths of the hydrocarbon radicals of the side and cross-linking groups.

Nanoregions in which the side-chain substituents of the polymer are concentrated are the sorption centers of the samples. Moreover, in accordance with the principle of similarity, it is quite expected that the solubility coefficient increases in the following series: nitrogen < methane < ethane < butane, in which the affinity of gas molecules for the side C10 substituents increases. Thus, based on WAXS data, one would expect a linear increase in solubility coefficients with the increasing concentration of side-chain substituent domains in the polymer. However, in the case of hydrocarbon cross-links, the solubility coefficients of ethane and n-butane for the C10-DdD sample are lower than those of C10-DD. Despite the high concentration of the side substituent domains, an increase in the length of the hydrocarbon cross-link leads to the formation of “defects” at the cross-link sites. Indirectly, this is confirmed based on a decrease in the density and apparent complete cross-linking of the polymer.

The transition from hydrocarbon to siloxane cross-linking reduces the solubility coefficients. Thus, when comparing C10-DdD and C10-Sil500, one can notice that the latter has solubility coefficients 1.2–2 times lower. In contrast, the transition to combined cross-linking leads to an increase in solubility coefficients. The positive point is that the nitrogen solubility coefficient barely changes with the increasing length of the siloxane cross-linking agent in the polymer. The solubility selectivity of the studied hydrocarbons in relation to nitrogen has trends similar to the solubility coefficients. Apparently, the solubility of these polymers is directly related to the packing density of side substituents in the polymer. It can be noted that the use of rigid cross-links, the sizes of which differ from the size of the side substituent, leads to a decrease in the selectivity of n-butane/nitrogen dissolution. The use of combined cross-linking based on high-molecular PDMS and 1,7-octadiene makes it possible to increase the selectivity of dissolution not only due to the organization of side substituents but also due to the high sorption capacity of PDMS itself [39].

Permeability coefficients, according to the basic law of transfer through non-porous membranes (the “dissolution-diffusion” mechanism), are the product of the diffusion coefficient and the corresponding dissolution coefficient. In this regard, it is worth considering the permeability coefficient as a macroproperty of the polymer, which includes a set of structural and physicochemical properties of the polymer. If we compare polydecylmethylsiloxanes cross-linked with diene hydrocarbons and siloxanes, it is worth noting that the nitrogen and methane permeability coefficients are higher for the latter. For ethane and n-butane, this trend is not so obvious. The sample C10-Sil25-OD is characterized by the highest permeability coefficient of ethane and n-butane, which emphasizes the decisive contribution of the sorption component required to transport through siloxane rubbers. Sample C10-OD has unexpectedly high permeability coefficients. Despite the high apparent cross-linking density (3.14·10^−4^ mol/g), this cross-linked sample is characterized by the lowest degree of crystallinity (5.7%), according to X-ray diffraction data. This is due to the formation of “defects” in the cross-linking region, since the length of the cross-link consisting of 8 CH_2_ groups (11.9 Å) is less than the length of the decyl substituents (13.8 Å). Such “defects,” for example, are not observed for sample C10-DD, in which the cross-link length is 14.4 Å. This sample demonstrates the lowest values of permeability coefficients for nitrogen and methane (gases with a low solubility coefficient) due to the difficult diffusion of these gases through the polymer.

If we trace the influence of the length of the hydrocarbon cross-link on the gas permeability of C10 films, we can note that there is a decrease in the permeability coefficients for ethane and n-butane and the corresponding selectivity with the increasing length of the cross-linking diene. When moving from hydrocarbon cross-linking to siloxane cross-linking with a comparable cross-linking agent length (samples C10-DD and C10-Sil500), the permeability coefficients increase due to the mobility of the siloxane chain, which led to an almost 3-fold increase in diffusion coefficients. However, the permeability selectivity of the C10-Sil500 sample is due to the lower solubility of hydrocarbons in this sample: the n-butane/nitrogen selectivity decreases from 77 (C10-DD) to 63 (C10-Sil500). The transition to combined cross-linking C10-Sil25-OD leads to increases in the permeability coefficients, recording the highest values of permeability coefficients in the series studied. This effect is obviously associated with the contribution of the high-molecular cross-linking of PDMS, which is characterized by high gas permeability, including hydrocarbons [19]. A slight increase in selectivity compared to C10-Sil500 (from 63 to 68 for n-butane/methane) is apparently due to the addition of 1,7-octadiene to PDMS during cross-linking. Indeed, it is cross-linking with 1,7-octadiene that makes it possible to provide a polymer structure that leads to its high selectivity for hydrocarbons: 3.7, 13.6, and 104 for methane, ethane and n-butane, respectively, with respect to nitrogen.

## 4. Conclusions

In this work, the complex influence of the type and length of the cross-linking agent on the structure and transport properties of C10 was studied for the first time. It has been shown that the use of a hydrocarbon or siloxane cross-linking agent has a different effect on the apparent cross-link density and the ordering of side decyl substituents in the polymer. In particular, the apparent cross-link density in the case of diene cross-linking is significantly higher than that in the case of polysiloxane cross-linking—with a length comparable to that of the cross-linking agent for the C10-DdD sample, the apparent cross-linking density was 2.35·10^−4^ mol/g, and for C10-Sil500, it was 1.49·10^−4^ mol/g. It can be noted that increase in the cross-link length favors the ordering of the side groups of C10, which is via in low-temperature X-ray diffraction. Short hydrocarbon diene cross-linking sterically hinders the mobility of side groups due to the rigidity of the cross-linking agent chain and leads to low values for the crystallinity degree of decyl substituents’. It is worth noting that the highest sorption selectivity is achieved for the C10-Sil25-OD sample with combined cross-linking. Apparently, such a combination of cross-linking agents (short hydrocarbon and long polysiloxane) not only ensures the high availability of the dynamic free volume of the polymer for sorption but also makes it possible to realize the properties of a number of side substituents.

The value of the permeability coefficient is of practical importance since it is directly related to mass transfer through the membrane, and it is a superposition of the diffusion and dissolution of gas in the polymer material. C10 hydrocarbon cross-linking leads to lower gas permeability coefficients for gases for which transfer occurs, mainly due to the diffusion component (nitrogen, methane) compared to polysiloxane cross-linking. For ethane and n-butane, there is no such clear dependence since the sorption component makes a decisive contribution to their transfer through membranes. However, if we consider the value of hydrocarbon/nitrogen selectivity, it becomes obvious that hydrocarbon cross-linking makes it possible to better realize the selectivity of C10 towards hydrocarbons compared to polysiloxane cross-linking. The most selective sample is C10-OD (n-butane/nitrogen-104), and it is the sample most permeable to hydrocarbons among diene cross-linking agents. Based on the dissolution diffusion mechanism, the permeability coefficient is the product of the diffusion coefficient and the permeability coefficient. It is known that silicone rubbers are characterized by the preferential dissolution of hydrocarbons compared to nitrogen. This determines the order of magnitude of selectivity for the hydrocarbon/nitrogen pair. However, in the series of related polydecylmethylsiloxanes, the difference in the permeability selectivity of the hydrocarbon/nitrogen pair is precisely determined by the selectivity of diffusion of these materials. In the case of cross-linking with 1,7-octadiene, such a packing of side substituents is ensured, which allows the most selective diffusion of hydrocarbons in relation to nitrogen in the studied series. In this regard, we can argue that it is cross-linking with 1,7-octadiene that allows better separation under ideal conditions. Thus, the fundamental influence of the type of cross-linking agent on the transport and separation properties of C10 has been demonstrated.

## Figures and Tables

**Table 1:** Membrane abbreviation, types and concentrations of the cross-linking agent (CA).

Membrane Abbreviation	Cross-Linking Agent (CA)	Ratio 1-Decene/CA, mol/mol
C10-OD	1,7-octadiene	20
C10-DD	1,9-decadiene	20
C10-DdD	1,11-dodecadiene	20
C10-Sil500	PDMS (M_n_ = 500 g/mol)	15
C10-Sil25-OD	PDMS (M_n_ = 25,000 g/mol) 1,7-octadiene	15

**Table 2:** Melting temperature (T_m_) and melting enthalpy (ΔH) for cross-linked C10.

Membrane Abbreviation	T_m_, °C	ΔH, J·g^−1^
C10-OD	−39.2	−26.7
C10-DD	−41.5	−20.9
C10-DdD	−40.9	−22.8
C10-Sil500	−43.3	−22.0
C10-Sil25-OD	−38.9 ^1^ −44.0 ^2^	−28.6

^1^ Presumably for C10 crystallites. ^2^ Presumably for PDMS crystallites.

**Table 3:** WAXS data for comb-like polysiloxanes at –196 °C (d_1_, d_2_, and d_3_—distances obtained via mathematical decomposition into plateau peaks; d_m_—interplanar distances corresponding to the crystallization of side chains).

Membrane Abbreviation	d_1_, Å	d_2_, Å	d_3_, Å	d_m_, Å	Crystallinity Degree of Side-Chain Substituents (*α*), %
C10-OD	16.2	8.0	5.6	4.1	5.7
C10-DD	14.7	7.7	-	4.1	6.2
C10-DdD	18.1	8.6	7.0	4.1	6.9
C10-Sil500	-	9.7	6.1	4.0	7.1
C10-Sil25-OD	-	7.1	-	4.1	7.6

**Table 4:** Cross-linking density, density of obtained polymers, and their sorption and swelling in toluene.

Membrane Abbreviation	Sorption, g/g	Swelling, cm^3^/cm^3^	Density, g/cm^3^	Apparent Cross-Linking Density, 10^−4^ mol/g
C10-OD	0.862 ± 0.009	0.928 ± 0.020	0.985 ± 0.096	3.14 ± 0.06
C10-DD	1.221 ± 0.007	1.382 ± 0.016	0.966 ± 0.097	2.44 ± 0.06
C10-DdD	1.262 ± 0.006	1.490 ± 0.024	0.928 ± 0.093	2.35 ± 0.17
C10-Sil500	2.749 ± 0.009	2.892 ± 0.018	0.929 ± 0.093	1.49 ± 0.09
C10-Sil25-OD	2.577 ± 0.007	2.461 ± 0.029	0.922 ± 0.092	1.79 ± 0.04
C10-Sil25 *	3.752 ± 0.005	3.287 ± 0.031	0.929 ± 0.093	1.38 ± 0.02

* Additional cross-linked using only the PDMS (M_n_ = 25,000 g/mol) sample for comparison.

**Table 5:** Diffusion coefficient and selectivity of cross-linked C10.

Membrane	Diffusion Coefficient, 10^−8^ cm^2^/s				Diffusion Selectivity (X/N_2_)		
	N_2_	CH_4_	C_2_H_6_	C_4_H_10_	CH_4_	C_2_H_6_	C_4_H_10_
C10-OD	520 ± 26	490 ± 25	260 ± 13	160 ± 8	1.0 ± 0.10	0.5 ± 0.05	0.3 ± 0.03
C10-DD	230 ± 10	180 ± 9	120 ± 6	55 ± 3	0.8 ± 0.08	0.5 ± 0.05	0.2 ± 0.02
C10-DdD	260 ± 10	200 ± 10	110 ± 6	55 ± 3	0.8 ± 0.08	0.4 ± 0.04	0.2 ± 0.02
C10-Sil500	660 ± 30	490 ± 25	240 ± 10	135 ± 7	0.7 ± 0.07	0.4 ± 0.04	0.2 ± 0.02
C10-Sil25-OD	1010 ± 50	420 ± 20	220 ± 10	125 ± 6	0.4 ± 0.04	0.2 ± 0.02	0.1 ± 0.01

**Table 6:** Solubility coefficient and selectivity of cross-linked C10.

Membrane	Solubility Coefficient, 10^−2^ cm^3^/(cm^3^cm.Hg)				Solubility Selectivity (X/N_2_)		
	N_2_	CH_4_	C_2_H_6_	C_4_H_10_	CH_4_	C_2_H_6_	C_4_H_10_
C10-OD	0.1 ± 0.01	0.4 ± 0.02	2.8 ± 0.14	34 ± 2	3.9 ± 0.4	28 ± 3	344 ± 30
C10-DD	0.2 ± 0.01	0.7 ± 0.04	3.8 ± 0.20	62 ± 3	4.6 ± 0.5	25 ± 3	416 ± 40
C10-DdD	0.2 ± 0.01	0.7 ± 0.04	3.5 ± 0.18	57 ± 3	4.4 ± 0.4	23 ± 2	379 ± 40
C10-Sil500	0.1 ± 0.01	0.5 ± 0.03	3.0 ± 0.15	36 ± 2	4.6 ± 0.5	27 ± 3	327 ± 30
C10-Sil25-OD	0.1 ± 0.01	0.9 ± 0.05	5.3 ± 0.27	65 ± 3	7.8 ± 0.8	44 ± 4	542 ± 50

**Table 7:** Permeability coefficient and selectivity of cross-linked C10.

Membrane	Permeability Coefficient, Barrer				Permselectivity (X/N_2_)		
	N_2_	CH_4_	C_2_H_6_	C_4_H_10_	CH_4_	C_2_H_6_	C_4_H_10_
C10-OD	53 ± 3	194 ± 10	723 ± 40	5510 ± 280	3.7 ± 0.4	13.6 ± 1.4	104 ± 10
C10-DD	35 ± 2	123 ± 6	395 ± 20	3170 ± 160	3.6 ± 0.4	11.4 ± 1.1	92 ± 9
C10-DdD	39 ± 2	133 ± 7	340 ± 20	3000 ± 150	3.4 ± 0.3	10.2 ± 1.0	77 ± 8
C10-Sil500	75 ± 4	250 ± 10	712 ± 40	4780 ± 240	3.3 ± 0.3	9.5 ± 1.0	63 ± 6
C10-Sil25-OD	120 ± 6	400 ± 20	1180 ± 60	8200 ± 410	3.3 ± 0.3	9.8 ± 1.0	68 ± 7

## Data Availability

Data are contained within the article.
